# Brucellosis in camel, small ruminants, and Somali pastoralists in Eastern Ethiopia: a One Health approach

**DOI:** 10.3389/fvets.2024.1276275

**Published:** 2024-03-25

**Authors:** Abdullahi Adan Ahad, Bekele Megersa, Bedaso Mammo Edao

**Affiliations:** ^1^Department of Microbiology and Veterinary Public Health, College of Veterinary Medicine, Jigjiga University, Jigjiga, Ethiopia; ^2^Department of Microbiology, Immunology and Veterinary Public Health, College of Veterinary Medicine, Addis Ababa University, Bishoftu, Ethiopia

**Keywords:** animals, brucellosis, occupationally linked humans, seroprevalence, Somali region

## Abstract

Brucellosis is a neglected bacterial zoonotic disease with economic and public health importance in pastoral communities of sub-Saharan Africa. A cross-sectional study was conducted from December 2021 to April 2022, to estimate the prevalence and identify the associated risk factors causing brucellosis in animals and associated with occupational diseases in humans from three selected districts of “the Somali Pastoral region,” Eastern Ethiopia. In this study, 1,000 serum samples were screened for anti-*Brucella* spp. antibodies using Rose Bengal Plate Test (RBPT) and further confirmed using a competitive enzyme-linked immunosorbent assay (cELISA). A structured questionnaire was used to collect the biodata of tested animals and animal attendants to test the association between explanatory and outcome variables. The overall animal level prevalence was 5% (95% CI, 6.1–7.2.0) in small ruminants, 2.9% (95% CI, 1.5–4.9) in camels, and 2.0% (95% CI, 0.2–3.7) in occupationally linked humans. Herd size and herd history of retained fetal membranes were risk factors associated with *Brucella* spp. seropositivity in animals (*p* < 0.05). Disposing of retained fetal membranes was significantly associated (*p* < 0.05) with *Brucella* spp. seropositivity in humans. Evidence of brucellosis in various livestock species and associated seropositivity in humans indicates the need for a coordinated One Health approach, considering sociocultural dynamics of pastoral communities in controlling brucellosis to safe guard public health and increase livestock productivity.

## Introduction

Brucellosis is one of the re-emerging bacterial diseases that posing public and animal health problems in many pastoral settings. Currently, 12 *Brucella* spp. are included in the genus *Brucella* (1), of which, *B. abortus* in cattle, *B. melitensis* in goats and camel, *B. suis* in pigs, *B. ovis* in sheep, *B. canis* in dogs, *and B. neotomae* in rats are considered as classical (2). The disease can be transmitted between animals and from animals to humans by direct contact or indirect contact with contaminated materials. Due to close physical contact with animals and the tradition of consumption of unpasteurized milk, pastoralists are at highest risk of contracting the disease (3).

Currently, three classical species, *B. abortus*, *B. melitensis*, and *B. suis*, have an essential impact on public health. Being a public health concern that poses economic losses, brucellosis is a devastating disease that lacks pathognomonic symptoms in humans (4), making it difficult to differentiate from febrile conditions including malaria (5). Annually, approximately 500,000 human infections have been reported every year in low-income and middle-income countries (LMICs), where livestock is their mainstay. In LMICs, the disease is endemic and remains neglected, with huge public and animal health-associated problems (6).

The risk factors that influence the transmission, maintenance, and/or control of animal brucellosis are related to livestock management practices, animal movements, environmental factors, pastoralist behaviors and practices, lack of veterinary control measures, socioeconomic factors, genetic content of the animal host population, and biology of *Brucella* spp. (7). Risk factors for human brucellosis include, but are not limited to, the handling of infected animals and ingestion of contaminated animal products such as unpasteurized milk and milk products (including cow, goat, and camel milk) and meat (8).

In humans, the disease can lead to long-term complications, disability, and reduced productivity, resulting in potential income loss. It also has a negative impact on livestock production, reducing milk production, causing infertility, abortion, and poor growth rates, leading to decreased profitability in the agricultural industry. The correct diagnosis of brucellosis presents difficulties as its symptoms in humans are non-specific and can resemble other diseases. Laboratory tests may yield false-positive or false-negative results, delaying proper diagnosis and treatment initiation (9).

*Brucella* spp. infection causes huge financial losses and community health concerns in countries around the world. Globally, the economic losses due to brucellosis are substantial. According to the Food and Agriculture Organization (FAO), the estimated annual economic losses caused by brucellosis in livestock production, including cattle, goats, and sheep, can range from USD 200 to 600 million. These losses result from decreased productivity, increased veterinary costs, trade restrictions, and losses in animal products. In addition to livestock-related losses, severe health-related problems in humans, including life-threatening conditions, should be taken into account when dealing with brucellosis socioeconomic impacts. These include healthcare costs, such as hospitalization, medication, and follow-up care, as well as productivity losses due to morbidity, disability, and potential income loss (10).

In Ethiopia, brucellosis is one of the top five prioritized zoonotic diseases in Ethiopia (11). The animal brucellosis was first reported in the 1970s (12). Since then, many seroepidemiological studies from different regions of the country showed a prevalence report that ranges from 1.5 to 22.2%. Most of these reports were either from limited livestock species or relatively confined in a single environmental setting. There are few studies conducted on the seroepidemiology of brucellosis in Somali pastoral regions, and those involving epidemiology of brucellosis and its public health significance at the human–animal interface are scarce. In addition, the magnitude of the disease in different livestock species sharing the same environmental settings is not well studied. Therefore, understanding the epidemiology of the disease in mixed livestock populations and pastoralists in Somali region is of paramount importance. Hence, the objectives of this study are (i) to estimate the seroprevalence; (ii) to identify brucellosis-associated risk factors for the disease in camel, small ruminants, and pastoralist herders; (iii) to assess knowledge, attitude, and exposure risks of the herders toward the disease.

## Materials and methods

### Description of the study area

**Figure 1:**
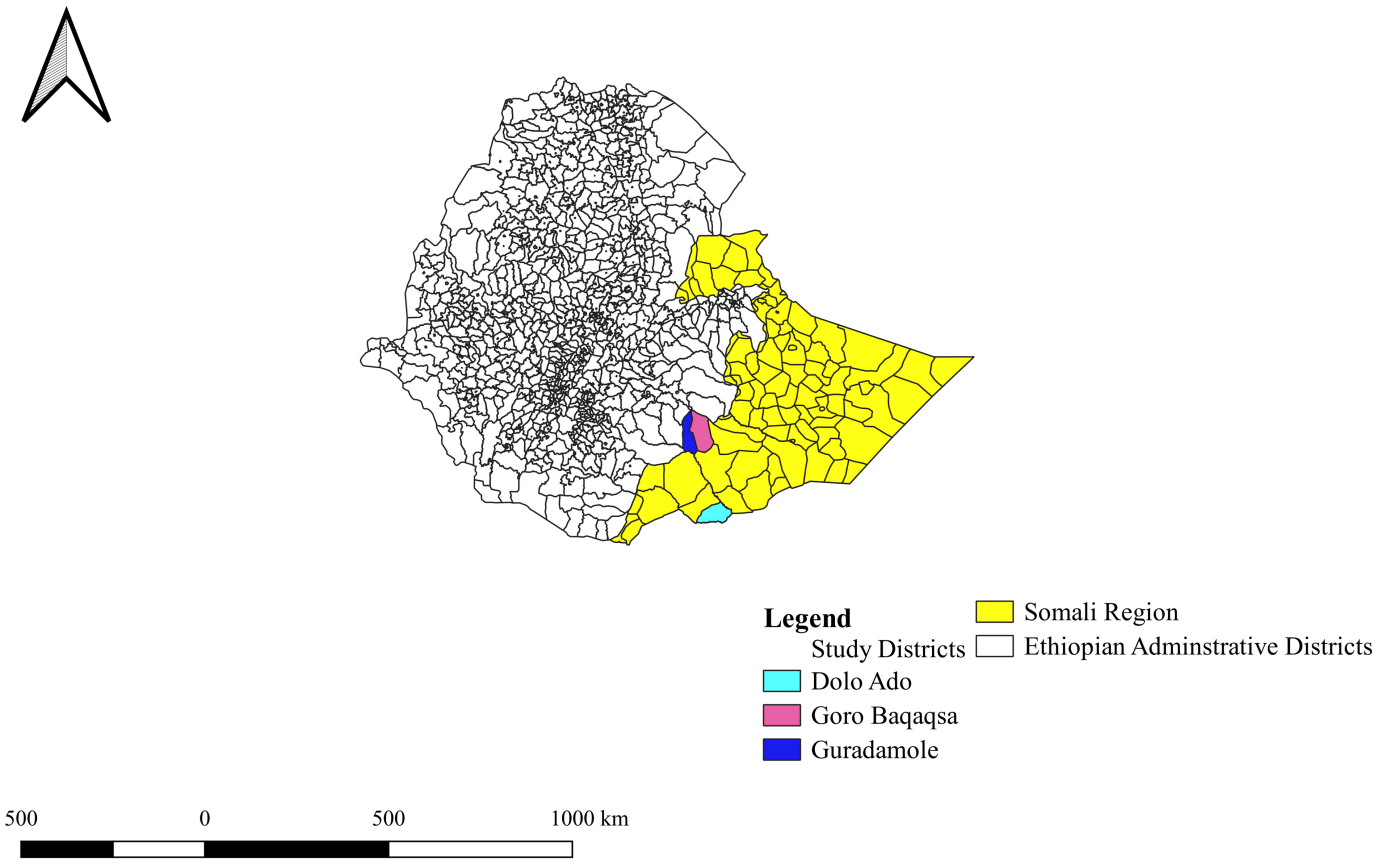
Map of the study areas. This map is extracted from Ethiopian shape file using QGIS version 3.20.0. Reproduced with permission of Ethiopian Mapping Agency.

This study was conducted in selected districts of the Somali region: Goro Baqaqsa, Guradamole, and Dolo Ado of Liban Zone (Figure 1). The Liban zone is 887 km away from Addis Ababa, Ethiopia. The communities are pastoralists, rearing livestock as a livelihood, and means of income. The climate varies from arid to semi-arid, which is characterized by regular water and fodder shortages, forcing pastoralists to seasonal migration with their animals. The altitude ranges from 250 to 1,500 m above the sea level and is located between 6°00′N 43°45′E. The area experiences average annual rainfall of 600–700 mm. The main rainy season, known as “Gu,” lasts from March to May, followed by the short dry season, known as “Xagaa,” which lasts from June to August. The short rainy season “Dayr” occurs between September and November, and the long dry season “Jilaal” occurs between December and March (13).

Pastoralists own large, mixed livestock species of herds, on which their daily livelihood depends from a social, economic, and dietary point of view. Many pastoralists in Ethiopia migrate seasonally with their animals in search of grazing land and water and share pastures and watering points. The livestock production system in the region is influenced by traditional pastoralist practices and the dry environment. Pastoralists lead to a nomadic lifestyle, constantly moving their animals to find food and water. Cattle, camels, sheep, and goats are the main livestock species, well adapted to the dry climate, and provide meat, milk, and income for pastoralist communities. However, the livestock production system faces challenges, such as inadequate market infrastructure, long travel distances to reach markets, and environmental and socioeconomic issues such as recurring droughts, land degradation, limited access to water, insufficient veterinary services, and conflicts over resources (14).

### Study design and study population

A cross-sectional study was undertaken from December 2021 to April 2022 to estimate the prevalence of brucellosis in animals and occupationally linked humans in the Somali pastoral region of Ethiopia. Animal-level bio-data were collected using a structured questionnaire that included sex, age, herd size, animal movement, parity, herd history of abortion and retained fetal membrane (RFM), and physiological status of the animals. Age was categorized into young (<6 months in goats and sheep and < 4 years in camels) and old (≥6 months in sheep and goats and ≥4 years in camels), herd size was considered as small (<50) and large (>50), animal migration (yes or no), number of parity (Null, ≤3 and > 3), herd history of abortion and RFM (Yes or No), and finally, physiological status of the animal was classified as dry, lactating, and pregnant. Furthermore, to assess the public health impact and estimate the magnitude of the disease in occupationally associated humans, blood samples were also collected from the owners of the animals, and information such as gender, age, the habit of consuming raw milk, assisting calving/birthing, and disposing of aborted fetuses and fetal material was recorded.

### Sampling method and sample size determination

In this study, a multistage sampling combined with the convenient sampling strategy was employed for sampling of individual animal species. Three districts were purposively selected based on livestock populations and proximity to the road. Two pastoralist associations (PAs) were randomly selected from each district, resulting in six PAs being included in the study. Within each selected PA, households were then selected based on the presence of more than two livestock species per household. This method ensured that the households were chosen more likely to have a diverse range of livestock. As a result, 60 households were included in the study. To gather data from these households, a further sampling process was conducted. From each household, a minimum of four people were randomly selected and sampled. Furthermore, a lottery system was used to select an individual animal from a herd, by assigning a number 1 (to be selected) or 0 (not to be selected) to an animal.

The number of animals in each household was considered as a herd and was sampled using a systematic random sampling technique. Sample size was determined using the expected prevalence of 7.5% in camels (11), 9.7% in sheep (15), and 16.5% in humans (12), by considering a 5% desired precision at a 95% confidence interval according to the formula previously published (16). Accordingly, 450 samples from camel, 300 samples from small ruminants, and 250 samples from humans were collected from the three districts.

### Sample collection and laboratory analysis

#### Blood sample collection

To minimize error, a bar code system was developed for both human and animal samples. The code is an abbreviation that consists of the first letter of the region, zone, district, and PAs (SLGBB0001). A specific label was fixed to the vacutainer tube after blood collection. After restraining the animals properly and having disinfected the area of venipuncture with 70% alcohol, 10 mL and 4–5 mL of blood were drawn from the jugular veins of camels, sheep, and goats, respectively. The blood samples were then labeled and left tilted overnight at room temperature to allow for clotting. Sera were later decanted into sterile cryovials. For human samples, approximately 5 mL of blood was drawn by a qualified nurse at the PAs via venipuncture of the medium cuboidal vein using a plain vacutainer tube. The sera samples were then transported to Jigjiga Regional Veterinary Diagnostic and Research Laboratory in an ice box and stored at −20°C for further processing.

#### Serological test

##### RBPT

The serum samples were screened for anti-*Brucella* antibodies using RBPT, according to the standard procedure described by Nielsen (17). Any visible agglutination was considered positive. Based on the level of clumping, the results were read as weak, moderate, and strong agglutinations. For interpretations of the results, both positive and negative control sera were used as recommended by OIE (18). For the test, 30 μL of RBPT was used in camel. To improve the sensitivity of RBPT, one volume of antigen and three volumes of serum (e.g., 25 μL with 75 μL) were used in sheep and goats as recommended by Garin-Bastuji et al. (19). The antigen and test serum were thoroughly mixed using a plastic applicator for 4 min, and the result (presence of agglutination or not) was read immediately (18).

##### Competitive ELISA

All the RBPT-positive animal and human sera samples were further tested at Jigjiga Veterinary Diagnostic and Research Laboratory using a commercial cELISA (Abbexa Ltd., Cambridge Science Park, and Cambridge, CB4 0EY, United Kingdom) and an IgG ELISA (Abbexa LTD, Cambridge, UK), respectively, following the manufacturer’s protocol.

##### Case definition

A sample was considered seropositive when it tested positive for RBPT and cELISA methods. A flock or herd was considered seropositive when at least one animal tested positive for both tests. Since vaccination against brucellosis is not practiced in Ethiopia, seropositivity in this study was considered to be due to natural infection.

### Data analysis

The data from the field and laboratory were entered into Microsoft Excel and analyzed using R software version R-3.3.0. Univariate logistic regression model was used to determine putative risk factors associated with *Brucella* spp. seropositivity. Multiple logistic regression was used to model the relationship between a binary dependent variable (Result) and multiple independent variables (age, sex, species, RFM, parity, abortion, physiological status, flock/herd size, and migration). The process involved data preparation, model specification, model estimation using maximum likelihood estimation, and interpretation of results through estimated coefficients and *p* > 0.2. As some variables that are individually insignificant but could potentially be significant in multivariable analysis, a cutoff value of *p* was inflated to a value ≤0.2. Multiple logistic regression allows for understanding the relationships between the dependent variable and multiple independent variables, accounting for interactions and potential confounding effects. For variables that showed strong colinearity (*p* < 0.05), one of the two variables was excluded based on biological plausibility to *Brucella* infection. Stepwise backward elimination procedure was employed for the selection of variables in the final model. The strength of association of exposure variables with seropositivity of the disease was assessed using odd ratios.

## Results

### Descriptive statistics of seroprevalence

A total of 450 samples from camel, 300 samples from small ruminants, and 250 samples from humans collected from the three districts were tested for anti-Brucella antibodies. The overall seroprevalence was 5% (95% CI = 6.1–7.2.0) in small ruminants, 2.9% (95% CI = 1.5–4.9) in camel, and 2.0% (95% CI = 0.2–3.7) in occupationally linked humans. The highest seroprevalence of 6.5% (95% CI = 3.5–10.8) was observed in goats compared with camels 2.9% (95% CI = 1.5–4.9) and sheep 2.0% (95% CI = 0.2–7.1). Regarding districts, the overall seroprevalence of the disease in livestock was lowest in Dolo Ado (3.2%) compared with the other two districts with 4% in each.

The distribution of seroreactor animals and humans varied among the three districts. Goro Baqaqsa district had the highest proportion of seropositive sheep and goats (6, 95% CI = 2.2–10) and humans (2.8, 95% CI = 0.3–9.6) compared with the other districts. Dolo Ado had the highest (3, 95% CI = 1.1–7.6) seropositive camels compared with the other two districts. However, it had the lowest seroprevalence in humans (1, 95% CI = 0.02–5.4) and sheep and goat (3, 95% CI = 0.6–8.5). When pastoral village was considered, seropositive animals were found in 83% (5/6) of the villages. Village level seropositivity was more frequently detected in sheep and goats (5%) than in camel (2.9%). The seroprevalence ranges from 0 to 9.8% in sheep and goats and from 0 to5.9% in camels (Supplementary Table S1).

### Risk factors for *Brucella* spp. seropositivity in sheep and goats

The major variables that were considered in the univariable logistic regression analysis include district, sex, species, age, herd size, animal mobility or migration, parity, physiological status, history of abortion, and retained fetal membranes. The result showed that herd history of RFM was significantly associated with *Brucella* spp. seropositivity (*p* < 0.05; Table 1).

**Table 1:** Univariable logistic regression analysis for brucella seropositivity in small ruminants.

Variables	Category	Samples	No positive (%)	OR (95% CI)	Value of *p*
	Dolo Ado	100	3 (3)	Ref	
Districts	Goro Baqaqsa	100	6 (6)	2.0 (0.5–8.5)	0.32
	Guradamole	100	6 (6)	1 (0.4–3.3)	1.00
Species	Ovine	99	2 (2)	Ref	
	Caprine	201	13 (6.5)	0.9 (1–21)	0.21
Sex	Male	137	3 (2.5)	Ref	
	Female	163	12 (7.4)	3.5 (1.0–12.8)	0.05
Age	Young	121	4 (3.3)	Ref	
	Adult	179	11 (6.1)	2 (0.6–7)	0.27
Flock size	Small	156	5 (3.2)	Ref	
	Large	144	10 (6.4)	3.14 (1–11)	0.05
Migration	No	177	8 (6.8)	Ref	
	Yes	83	7 (3.8)	0.96 (0.3–3.0)	0.94
Parity	>3	59	3 (5.1)	Ref	
	≤3	104	9 (8.7)	1.43 (0.3–4.6)	0.53
Abortion	Not aborted	128	4 (3.1)	Ref	
	Aborted	35	8 (23)	3 (0.9–9.7)	0.08
RFM	No	134	2 (1.5)	Ref	
	Yes	29	10 (33.3)	8.2 (2.4–29)	**0.00** ^*^
PS	Lactating	38	2 (5.2)	Ref	
	Dry	84	6 (7.1)	0.4 (0.02–2.0)	0.40
	Pregnant	41	4 (9.8)	1.4 (0.4–4.6)	0.46

Reference category: ^*^Statistically significant. PS, physiological status; RFM, retained foetal membrane.

In the final multivariable logistic regression model, all variables with a value of p less than or equal 0.2 on the univariate analysis were included. The result indicated that small ruminants from a large herd were 5.01 times more likely to acquire *Brucella* spp. infection compared with those kept in a small herd (OR: 5.01, 95% CI: 1.2–21.4, *p* = 0.02). Similarly, sheep and goats with a history of retained fetal membranes were more likely to be seropositive for *Brucella* spp. infection than sheep and goats without a history of retained fetal membranes (OR: 9, 95% CI: 1.9–42; Table 2).

**Table 2:** Multivariable logistic regression analysis for brucella seropositivity in small ruminants.

Variable category	Category	Samples	No positive (%)	OR (95% Cl)	Value of *p*
Flock size	Small	156	5 (3.2)		
	Large	144	10 (6.4)	5.01 (1.2–21.4)	**0.02** ^*^
Abortion	Not aborted	128	4 (3.1)	Ref	
	Aborted	35	8 (23)	3 (0.9–9.7)	0.08
RFM	No	134	2 (1.5)		
	Yes	29	10 (33.3)	8.2 (2.4–29)	**0.00** ^*^

^*^Statistically significant.

### Risk factors for *Brucella* spp. seropositivity in camels

The univariable logistic regression analysis indicated that seropositivity in camels was significantly associated with pregnancy (OR = 3.3, 95% CI: 1.4–15, *p* < 0.05), history of abortion (OR = 3.9, 95% CI: 1.2–13, *p* < 0.05), and RFM (OR = 22, 95% CI: 5.9–8, *p* < 0.05; Table 3). Multivariable logistic regression model using variables with value of *p* ≤ 0.2 from univariate analysis indicated that history of RFM had a significant association with *Brucella* spp. seropositivity (95% CI: 12.7–60, *p* < 0.05; Table 4).

**Table 3:** Univariable logistic regression analysis for brucella seropositivity in camel.

Variables	Category	Samples	No positive (%)	OR (95% CI)	*p*-value
Districts	Goro Baqaqsa	150	4 (2.7)	Ref	
	Guradamole	150	4 (2.7)	1.1 (0.2–4)	0.73
	Dolo Ado	150	5 (3.3)	1.2 (0.3–4.8)	1.0
Sex	Male	151	1 (0.7)	Ref	
	Female	299	12 (4.0)	0.2 (0–1.2)	0.07
Age	Young	256	5 (2.0)	Ref	
	Adult	194	8 (4.1)	2.2 (0.7–6.7)	0.18
Herd size	Small	162	3 (1.9)	Ref	
	Large	288	10 (3.5)	1.9 (0.5–7)	0.33
Migration	No	185	5 (2.7)	Ref	0.84
	Yes	265	8 (3.0)	1 (0.4–3.5)	
Parity	Null	159	4 (1.9)	Ref	
	≤3	124	7 (5.6)	2.3 (0.6–8)	0.18
	≥3	16	1 (6.3)	2.6 (0.3–24)	0.40
Abortion	Not Aborted	250	7 (2.8)	Ref	
	Aborted	49	5 (10.2)	3.9 (1.2–13)	**0.02** ^*^
RFM^**^	No	285	7 (2.5)	Ref	
	Yes	14	5 (35.7)	22 (5.9–8)	**0.00** ^*^
Physiology	Dry	200	4 (2)	Ref	
	Pregnant	51	5 (9.8)	5.3 (0.7–20)	**0.01** ^*^
	Lactating	48	3 (6.3)	3.3 (1.4–15)	0.13

^*^Statistically significant. ^**^Retained fetal membranes.

**Table 4:** Multivariable logistic regression for brucella seropositivity in camel brucellosis.

Variables	Category	Samples	No positive (%)	OR (95% CI)	*p*-value
Age	Young	256	5 (2.0)		
	Adult	194	8 (4.1)	1.7 (0.1–31)	0.709
Parity	Null	159	4 (1.9)		
	≤3	124	7 (5.6)	0.4 (0.0–3.2)	0.350
	≥3	16	1 (6.3)		
Abortion	Not aborted	259	7 (2.8)		
	Aborted	49	5 (10.2)	0.7 (0.1–5.6)	0.80
RFM	No	285	7 (2.5)		
	Yes	14	5 (35.7)	35 (12.7–60,)	**0.004** ^*^
PS	Lactating	48	3 (6.3)		
	Dry	200	4 (2)		
	Pregnant	51	5 (9.8)	1.6 (0.5–4.6)	0.43

^*^Statistically significant.

### Serological survey for human brucellosis

Higher seroprevalence was observed in female individuals, 2.5% (*n* = 4) compared with male, 1.1% (*n* = 1). Participants from households with seropositive animals had seven times more risk of being seropositive for *Brucella* spp. infection than those without seropositive animals (OR = 7, 95% CI: 0.2–13.7, *p* = 0.72). Participants who consumed raw milk had 5.7 times higher odds of *Brucella* spp. seropositivity compared with those who consumed pasteurized milk (OR = 5.7; 95% CI = 0.9–35, *p* = 0.06); however, this was not statistically significant. Individuals who assisted during calving had higher odds of *Brucella* spp. seropositivity than those who had not higher odds of *Brucella* spp. seropositivity (OR: 3.6, 95% CI: 0.58–22, *p* = 0.16), and this was not statistically significant. The multivariable logistic regression analysis indicated that handling and disposing of aborted fetal materials was significantly associated with *Brucella* spp. seropositivity in humans (95% CI: 4.0–45, *p* = 0.001; Tables 5, 6).

**Table 5:** Univariable logistic regression analysis for brucella seropositivity in humans.

Variables	Categories	Samples	No positive (%)	OR (95% CI)	*p*-value
Districts	Dolo Ado	100	1 (1)	Ref	
	Goro Baqaqsa	72	2 (2.7)	1.4 (0.2–10.0)	0.74
	Guradamole	78	2 (2.6)	0.6 (0.1–7.0)	0.50
Gender	Male	89	1 (1.1)	Ref	
	Female	161	4 (2.5)	7.5 (0.8–68)	0.07
Age	18–60 years	177	1 (0.6)	Ref	
	<18 years	37	1 (2.7)	0.2 (0.1–3)	0.25
	>60 years	36	3 (8.3)	5.0 (0.3–87)	0.33
Seropositive animals at household	No	196	2 (1.0)	Ref	
	Yes	54	3 (1.9)	7 (0.2–13.7)	0.72
Consume raw milk	No	59	1 (1.7)	Ref	
	Yes	191	4 (2.1)	5.7 (0.9–35)	0.06
Ass. Calving	No	210	3 (1.4)	Ref	
	Yes	40	2 (5)	3.6 (0.58–22)	0.16
Dispose RFM	No	234	2 (0.9)	Ref	
	Yes	16	3 (19)	26 (4.0–45.6)	**0.001** ^*^

^*^Statistically significant.

**Table 6:** Multivariable logistic regression analysis for brucella seropositivity in humans.

Variables	Categories	Samples	No positive (%)	OR (95%Cl)	*p*-value
Districts	Goro Baqaqsa	72	2 (2.7)		
	Guradamole	78	2 (2.6)	2.6 (0.4–14)	0.26
	Dolo Ado	100	1 (1)		
Gender	Male	89	3 (3.4)		
	Female	161	2 (1.2)	14 (0.8–25.5)	0.06
Consume	No	159	2 (3.7)		
Raw milk	Yes	191	3 (1.5)	2.5 (0.9–68)	0.56
Assist birthing or	No	210	3 (1.4)		
Calving	Yes	40	2 (5)	8 (0.6–127)	0.11
Dispose RFM	No	234	2 (0.8)		
	Yes	16	3 (23)	37.4 (5.1–18.5)	**0.001** ^*^

^*^Statistically significant.

## Discussion

This study showed an overall brucellosis seroprevalence of 5% (95% CI: 6.1–7.2) in camel, sheep, goats, and humans in three districts of Somali region, Ethiopia. Two tests were used serially to rule out false-positive cross-reactions and maintain maximal specificity (12, 13). A combination of RBPT and c-ELISA was used to test camel and human sera, whereas modified mRBPT and c-ELISA were used to test sera samples from sheep and goats. RBPT was used as a screening test because of its high sensitivity (20). Competitive ELISA was used by its high specificities to exclude false-positive cross-reactions. False-positive serological reactions in RBT could be due to cross-reactions with smooth lipopolysaccharide (S-LPS) antigens of other gram-negative bacteria. As there has never been history of vaccination in Ethiopia, seropositivity in all cases is due to natural infection (15, 21).

In this study, the animal level seroprevalence of 5% detected in small ruminants was comparable with the report by Traoré et al. (22) in Mali, who reported a prevalence of 4.1%. However, the prevalence estimated in the current study is higher than a prevalence with the report of 3.33% by Dosa et al. (23) from Southern Nation Nationalities and People (SNNP) region in Kolme and Abala Abaya districts, 0.24% by Geletu et al. (24) from Eastern Hararge, Oromia Region, and 0.9% by Girmay et al. (25) from sheep export farm in Adama, and 0.4% by Yeshwas et al. (26) from Bahir Dar. On the other hand, a higher prevalence of 12.35 and 13.7% than the present study was reported by Tegegne et al. (27) and Tedeg et al. (28) in Afar pastoral region, respectively. The observed differences in seroprevalence might be due to variation in the sensitivity and specificity tests used, geographic location, and sample size.

In this study, larger herd/flock size was found to have a higher seroprevalence (3.5%) than a smaller herd size (2.5%). This is in agreement with the findings by Traoré et al. (29) who reported 6.9 and 4.8%, respecively. However, this is inconsistent with the study by Rob et al. (30) who reported 19.35% in smaller and 6.45% in larger herds. This variation in the prevalence could be attributed to an increase in stock density in large herd sizes, which facilitates transmission of *Brucella* spp. infection during calving or abortion. Furthermore, this variation could be influenced by fluctuations in disease prevalence at the overall animal level and the herd size during the study period.

There was a significant association (*p* < 0.001) between *Brucella* spp. seropositivity and a history of RFM as previously reported (20, 29, 31). On the contrary, Deddefo et al. (32) reported that a history of RFM had no association with *Brucella* spp. seropositivity. The difference could be due to variations in physiological status of the studied population. However, this study is in agreement with Weken et al. (33) who reported that a history of retained fetal membranes was significantly associated with *Brucella* spp. seropositivity in small ruminants (*p* = 0.04). When abortion is caused by *Brucella* spp. infection, the placenta is frequently retained, and there is inflammation of the uterine wall (metritis). This explains that retained fetal membrane is a sequel of brucellosis (34).

In the current study, the overall animal level seroprevalence of 2.9% was detected in camels. This is similar to the reports of previous studies (23–26) conducted in similar agroecology in Ethiopia. Conversely, Bekele et al. (35) and Hadush et al. (36) reported a higher animal-level prevalence of 5.4 and 4.1%, respectively, in Afar pastoral region. The observed differences in seroprevalence could be due to differences in herd size, absence or presence of infectious foci, such as *Brucella-*infected herds, sample size, and sensitivity and specificity of tests used.

This study also showed that female camels had higher seroprevalence of brucellosis (4%) than males (0.7%). The same trend of a higher prevalence was observed in the report by Waktole et al. (37) with prevalence of 9.2 and 7% in female and male animals, respectively. This could be explained by the longer period in which female camels are kept in herds for breeding purposes compared with male camels. In the latter cases, camels are usually fed and sold off, except for few individuals that are kept for breeding purposes, haulage, and transport purposes (38). However, in this study, the findings are inconsistent with the report by Bekele et al. (35), who reported a higher prevalence in male camels than females. The differences in the proportion of male and female animals sampled could also contribute to the observed variations in the seroprevalence of camel brucellosis in different sexes.

In natural hosts, brucellosis is characterized by reproductive losses such as infertility, abortion, and birth of weak offspring (30, 39). In this study, seropositivity to *Brucella* spp. infection was significantly associated with RFM and history of abortion of camels, as previously reported in Ethiopia (24, 40). Abortion due to brucellosis is linked to the ability of the bacterium to adapt to the intracellular replicative niche typically characterized by low pH and reactive oxygen intermediates (41).

In general, seropositivity to *Brucella* spp. infection varied among different districts, animal species, and pastoral villages. This could be attributed to the difference in the herd size and sample size tested per visited households. Somali pastoralists move their livestock to different villages, districts, or even cross-national and international borders by travelling several kilometers in search of better pasture and watering points during short drought cycles driven by climate changes. This results in concentration of animals in specific areas, a factor that facilitates spillover of *Brucella* spp. from infected animals to susceptible populations. This, in turn, results in an emergence of new infectious foci contributing to the variability in seroprevalence of brucellosis among different villages and districts.

Wildlife and domestic animal population sharing same ecology in a traditional livestock production system was reported to be an important risk factor for transmission of brucellosis. The transmission of brucellosis between wildlife and domestic animals in a traditional livestock production system could occur by direct contact, as infected wildlife and the domestic counterparts may come into contact when sharing the same grazing areas. Additionally, environmental contamination can take place when infected wildlife shed *Brucella* spp. through bodily secretions such as urine, feces, or placental fluids, thus exposing domestic animals that come into contact with these contaminated sources (14, 33). In this study, at least two seroreactor animal species were identified in villages and households visited. Though the possibility of host-switching of *Brucella* spp. cannot be ruled out (42), particularly when different animal species mix so freely, the findings of the current study may suggest that *B. abortus* and *B. melitensis* circulate in this pastoralist population (43). This warrants more research studies, particularly molecular detection in the study areas to determine the prevalent *Brucella* spp. strains, which will also be essential before embarking on any vaccination program.

The overall prevalence of 2.0% was recorded in occupationally linked humans in the study area (*n* = 5/250; CI = 0.0–0.04), indicating the public health importance of the disease in this pastoral setting. This is comparable with the findings by Edao et al. (44), who reported 2.6% in Borana, and Ibrahim et al. (13), who reported 2.8% in the Somali region. However, Tschopp et al. (45) reported a higher prevalence of 48.3% in Afar and 34.9% in Somalia region. The difference observed could be attributed to the degree of endemicity of the disease in the livestock population, degree of exposure, sample size, the difference in location, variability related to the type of diagnostic test used, and the different time period when the studies were conducted.

Older participants had higher prevalence (8.3%) than middle aged (2.7%) and young (0.6%) people. This finding is in agreement with Yapi et al. (22) from Mali, who reported 9.5% in old age, 6% in middle age, and none in young people. The increase in seropositivity of participants in the old age group could be due to an increasing exposure risk associated with an increase in age (46). Because *Brucella* spp. are known to prefer the reproductive organs of female animals, particularly the placenta and aborted tissue, it is reasonable to assume that improper disposal and handling of aborted fetuses and fetal membranes would increase the risk of transmission (47). Individuals who had close contact with RFM while disposing had a 26-fold higher risk of acquiring brucellosis compared with those who did not. This finding fairly disagrees with the report by Edao et al. (44) probably because the number of respondents and the level of awareness in the study areas were different.

Brucellosis in humans was reported to be associated with the consumption of unpasteurized milk (29, 48). The practice of consumption of raw milk is common among Somali pastoral communities ascribed to a belief that milk would lose its nutritional contents when pasteurized. This study indicated that 76.4% (*n* = 191) of participants had consumed raw milk; however, this practice was not significantly associated with seropositivity. The large proportion of participants who consumed raw milk could therefore indicate a potential risk of acquiring zoonotic infections including brucellosis. Reproductive organs such as placenta are known to be a predilection site for *Brucella* spp. Assisting animals during calving or birthing could therefore increase the risk of infection (47). Multivariate logistic regression model indicated a significant association with *Brucella* spp. seropositivity practice of assisting during calving or birthing (OR = 8; 95% CI = 0.6–127). This finding is in agreement with previous studies conducted in Northern Tanzania by Cash-Goldwasser et al. (49) and in Kenya by Muturi et al. (50) that showed assisting calving or birthing would increase the risk of infection.

The source of infection of humans with *Brucella* spp. is often a close contact with infected animals (21, 25, 26). In light of this, the present study revealed that seropositivity in humans was seven times higher in households with seropositive animals compared with those without seropositive animals. This is in agreement with the report by Osoro et al. (47) in Kenya and Edao et al. (44) in Ethiopia.

In conclusion, the current study revealed that antibodies against *Brucella* spp. in camels, sheep, and goats, sharing the same ecological zone and occupationally linked pastoralists in Somali Region, Ethiopia. Herd size and history of RFM were found to be risk factors for brucellosis in animals. Contact with RFM was significantly associated with *Brucella* spp. seropositivity in humans. The recurrent drought in the region triggered by climatic changes that contributes to the mobility of mixed livestock population in search of feed, and water will likely continue to enhance the endemicity of the disease in the area. Extensive epidemiological studies involving One Health approach need to be undertaken to isolate and characterize circulating *Brucella* spp. among humans and livestock in the study area. This would help to identify the transmission dynamics of *Brucella* spp. among the traditional mixed livestock production system. In this study, existence of close contact between humans and animals in the pastoral community and wide prevalence of brucellosis in various livestock species remarkably indicated the potential risk of public health. Therefore, feasible control strategy of the disease in respect of pastoral community and the sociocultural status through One Health approach is highly recommended.

### Limitations

In some villages of the study area, pastoralists refused to consent to allow blood sample collection from their herds contending that this practice could impede productivity of their animals. Therefore, the desired sample size has not been reached, particularly human and camel sample. The number of districts and villages surveyed was limited to areas with less security concerns. Hence, the results are non-generalizable.

## Data availability statement

The original contributions presented in the study are included in the article/Supplementary material, further inquiries can be directed to the corresponding author.

## Ethics statement

The studies involving humans were approved by Somali Region Health Bureau with certificate (ref: SRHB-18-7738/2022). The studies were conducted in accordance with the local legislation and institutional requirements. Written informed consent for participation in this study was provided by the participants’ legal guardians/next of kin. The animal studies were approved by the Research Ethical Review Committee of Addis Ababa University College of Veterinary Medicine and Agriculture (AAU-CVMA) with certificate (ref: VM/ERC/21/02/142022). The studies were conducted in accordance with the local legislation and institutional requirements. Written informed consent was obtained from the owners for the participation of their animals in this study.

## Author contributions

AA: Conceptualization, Data curation, Formal analysis, Investigation, Methodology, Software, Writing – original draft. BM: Conceptualization, Data curation, Supervision, Writing – review & editing. BE: Writing – review & editing.
