# Supplementary material for: Brucellosis in camel, small ruminants, and Somali pastoralists in Eastern Ethiopia: a One Health approach
Source: Front Vet Sci. 2024 Mar 25;11:1276275. doi: 10.3389/fvets.2024.1276275 (PMC11002772; doi:10.3389/fvets.2024.1276275)
Supplement: Supplementary file 1 [file Table_1.DOCX]

**Table 1**: Distribution of seroreactor animals and humans among the three districts in Liban zone, Somali Region, Ethiopia.

| District | Species tested | N^o^ Sampled | N^o^ Positive (%) |
| --- | --- | --- | --- |
| Goro Baqaqsa | Sheep and Goats | 100 | 6 (6) |
|  | Camel | 150 | 4(2.6) |
|  | Human | 72 | 2(2.8) |
| Guradamole | Sheep and Goats | 100 | 6(6) |
|  | Camel | 150 | 4(2.7) |
|  | Human | 78 | 2(2.6) |
| Dolo Ado | Sheep and Goats | 100 | 3(3) |
|  | Camel | 150 | 5(3) |
|  | Human | 100 | 1(1) |
| Total | **Sheep and Goats** | 300 | 15(5) |
|  | **Camel** | 450 | 13(2.9) |
|  | **Human** | 250 | 5(2) |

**Table 2A:** Distribution of seroprevalence of *Brucella* antibodies in Sheep and goats among pastoral villages in the three studied districts of Liban a zone

| District | Village | Species | N^o^ Sampled | N^o^ Positive (%) |
| --- | --- | --- | --- | --- |
| Goro Baqaqsa | Biyo badan | Sheep and Goats | 67 | 6(8.9) |
|  | Koya | Sheep and Goats | 33 | 0(0) |
| Guradamole | Adeley | Sheep and Goats | 41 | 4(9.8) |
|  | Gobes | Sheep and Goats | 59 | 2(3.4) |
| Dolo Ado | Bur-amino | Sheep and Goats | 44 | 0(0) |
|  | Heleweeyn | Sheep and Goats | 56 | 3(5.4) |
|  | **Total** | **Sheep and goats** | **300** | **15(5)** |

**Table 2B:** Distribution of seroprevalence of *Brucella* antibodies in camels among pastoral villages in the three studied districts of Liban zone.

| District | Village | Species | N^o^ Sampled | N^o^ Positive (%) |
| --- | --- | --- | --- | --- |
| Goro Baqaqsa | Biyo badan | Camel | 129 | 4(3.1) |
|  | Koya | Camel | 21 | 0(0) |
| Guradamole | Adeley | Camel | 99 | 1(1) |
|  | Gobes | Camel | 51 | 3(5.9) |
| Dolo Ado | Bur-amino | Camel | 74 | 2(2.7) |
|  | Heleweeyn | Camel | 76 | 3(4) |
|  | **Total** | **Camel** | **450** | **13(2.9)** |
